# Georgia State Medical Association

**Published:** 1893-05

**Authors:** 


### *SOCIETY REPORTS.*

### GEORGIA STATE MEDICAL ASSOCIATION.

### PROCEEDINGS OF THE FORTY-FOURTH ANNUAL MEETING, HELD AT AMERICUS APRIL 19, 20 AND 21, 1893.

### First Day—Morning Session.

The association convened in the city hall and was called to order by the President, Dr. A. A. Smith, of Hawkinsville, at 10 a. m.

An "Address of Welcome" was delivered by Mayor A. S. Cutts, which was responded to on behalf of the association by Dr. Mark H. O'Daniel, of Macon.

The first paper read was the President's "Annual Address" by Dr. A. A. Smith.

President Smith said that Georgia had her boards of education, her boards of trade and her various other boards, but she had no board of health. The sanitary condition of her two million inhabitants, dwelling within the borders of 137 counties, should be looked after and cared for in an official way; and a board of health, composed of the best medical talent in the State should be established for that purpose. He knew of nothing that would be more conducive to the protection of the general health and prosperity of the people than the permanent establishment of a State Board of Health. He also recommended the permanent establishment of a State Board of Examination, and said no one should be allowed to practice medicine within the borders of the State until he shall have passed a rigid and satisfactory examination under this board, and received therefrom a certificate of proficiency. This would drive away quacks, and thereby not only protect the health and lives of the people, but also the honor and dignity of the profession. Furthermore, the curriculum of medical institutions in Georgia should be raised to a higher standard. Nothing less than the three course system should be tolerated, and each of these courses should be made broader, deeper and more comprehensive. With a State Board of Health and a State Board of Examination permanently established, and the curriculum of the Georgia medical schools raised to a higher standard, the profession would greatly accelerate the onward and upward progress of such a cause, and as it moved onward and upward, higher and higher, mantled with the glory of its past, and crowned with the splendor of its future God's truth would bloom upon its brow, and God's approbation rest upon its work.

DR. C. C. HART, of Cross Keys, read a paper entitled "Shot-Gun Prescriptions," in which he asked whether it was right to make what is known as "shot-gun" prescriptions. Personally he thought it was not right, because it increased the difficulty of prescribing, and made the therapeutic action of medicines more uncertain and consequently less safe for the patient. We should strive to make medicine an exact science, which we could never hope to do without exact knowledge, and how are we to know what effect a certain medicine has when it is combined with several other medicines, some of them perhaps of an antagonistic nature.

DR. J. I. DARBY, of Americus, contributed a paper entitled "Puerperal Septicæmia and its Treatment."

### FIRST DAY—AFTERNOON SESSION.

The first paper read was by Dr. J. M. Head, of Zebulon, entitled "Puerperal Eclampsia with a Report of Cases."

True eclampsia might differ from epilepsy clinically, but the paroxysm of each was essentially the same. We find in eclampsia occasionally premonitory symptoms which, if properly interpreted by the obstetrician, will put him upon his guard and sometimes enable him to ward off the attack. However, in a considerable number of cases the symptoms are so slight as to escape detection, and suspicion is not aroused until severe convulsions are on. Hence we find it is of practical importance to bear well in mind the most common of the symptoms which are associated with cerebral troubles, such as severe headache, either general or unilateral, spots before the eyes, dizziness, blindness, vertigo or impairment of the intellectual faculties which are occasionally observed. Again, we see symptoms manifested in restlessness, irritability of temper, stupor, general indisposition, œdema of the cellular or subcutaneous tissue, shown by swelling of the lower extremities, but this one symptom bears with it a much more serious import when it is observed as occurring in the upper extremities or a swollen face. These symptoms are sufficient to excite the most serious apprehensions and lead us to make examinations of their causes.

So eclampsia should be considered of a functional nature through reflex influences, as chorea is an occasional feature of pregnancy. For the purpose of elucidation the speaker mentioned the prominent disorders of the pregnant stage.

CONTAGIOUSNESS OF CONSUMPTION.

Dr. G. Hopkins, of Thomasville, read a paper on this subject. The speaker said he had joined the growing army which placed tuberculosis in the category of contagious diseases, and his experience with this disease during nineteen years of investigation in Thomasville, which place is a resort for consumptives, bore him out in his opinion, and made him a willing subject of the great and erudite Koch. He does not doubt but that all men, women and children at some time or times receive into their air passages the tubercle bacilli, but fortunately the great majority possessed the power of repelling them and throwing them off, they did not find that soil, so to speak, which is adapted to their growth. Indians in a state of nativity seemed impervious to the germs of consumption, but were now dying by thousands on the reservations. The whites and the blacks in prisons and asylums all over the world labored under similar conditions. A report from the Illinois State Prison at Joliet says that there are fourteen hundred convicts within the walls, and fully one-third of them have consumption in a light or bad form. Nearly all deaths of persons in the penitentiary have been caused by consumption.

Dr. Hopkins emphasized tho danger that lurks in sleeping cars, in carpets, bedding, clothing, and in the walls of apartments occupied by consumptives, which have not been properly renovated and rendered harmless by antiseptic measures.

Consumptives should be forced to provide for the destruction of sputa. Whenever situated so as not to expectorate directly into a germicide or the fire, they should use some means of conveying the sputa to the germicide flames. If handkerchiefs or clothes are used they should not be sent to the laundry, as human happiness and life are jeopardized through the probability of inoculation through abrasions upon the hands. These bacilli should never be allowed to dry up and impregnate the air, as is now done through ignorance of possible result. Numerous experiments by leading medical authorities have proved beyond doubt that consumption is an inoculable disease, and so rapidly is the throng of converts growing that the speaker would not be surprised if, even in his day, resorts now soliciting the patronage of the consumptive will be quarantining against him.

DR. J. MCFADDEN GASTON, of Atlanta, read a paper entitled "Science in Medicine and Surgery." (Published in this issue.)

DR. MARK H. O'DANIEL, of Macon, read a short paper on "Multiple Neuritis, Alcoholic," and reported a case. The patient was thirty-six years of age, fleshy, short build, short neck, florid complexion, a bilious temperament, and a bookkeeper by occupation. The patient had taken but little exercise for quite a long while except in the elbow joints. He came of a nervous ancestry, an aunt having died of climacteric mania, and other relatives who had been insane for a short time, showing beyond doubt a predisposition on the part of the patient to nervous trouble, which if it had not existed, the use of alcohol might not have precipitated the disease in question. The patient drank from one to two quarts of whiskey a day, that is, during twenty-four hours. He had all the symptoms of such cases. Under appropriate treatment the patient made a good recovery.

DR. WILLIS F. WESTMORELAND, of Atlanta, followed with a paper entitled "Syphilis from a Sociological Standpoint," in which he said, year after year, particularly in the clinics, syphilitic subjects, a great many of them women, came before him suffering from this disease in all its stages, from the original lesion to the worst ravages of the second and tertiary stages, with mucous patches and syphilitic ulcers covering them. And when he found, upon investigation, many of them servants, occupying various household positions as chambermaids, nurses, cooks, waitresses, etc., he could not but think that God protects the family exposed to the baleful contamination of their service, and particularly the poor little defenceless ones whom they nurse, and with whom their relations are so intimate. Any one who has not investigated the subject will be overwhelmed with surprise at the number of cases acquired otherwise than by sexual intercourse, and the various means by which it has been transmitted. Physicians and dentists are themselves exposed, and likewise expose their patients to these dangers. Thousands of physicians and midwives in the practice of their calling, in examining and operating on syphilitic subjects, or by post mortem, are exposed. Dentists by having their fingers scratched against a rough tooth or instrument, while working on a patient whose mouth is filled with mucous patches. On the other hand, many more patients are infected by the carelessness of physicians.

SECOND DAY—MORNING SESSION—April 20th.

DR. W. H. ELLIOTT, of Savannah, addressed the association on "Elastic Constriction as a Hæmostatic Measure," his remarks being a review of Senn's method. He said the association was familiar with Esmarch's method, which consisted in putting on an elastic bandage from the periphery slowly and gradually so as to empty the limb of blood, putting it on up to a point just above where the surgeon is going to operate. He then puts on a narrow elastic band at that point, so as to cut off the circulation from the limb entirely, then, removing the compression bandage, to go on with the operation, which he had fitly called a "bloodless operation." Esmarch was not the inventor of the bloodless operation, but surgeons were indebted to his genius for improving its technique and for making this established method a popular procedure in surgery. The speaker then stated the two grave objections against the method as used by Esmarch, as advanced by Dr. Senn. The first objection was that forcible compression of the blood out of the tissues by an elastic bandage was liable to send into the surrounding tissues the elements of microbic and malignant diseases. Cancer cells may thus be scattered and disseminated through the system, or the cells of pus or of tuberculosis might be sent abroad to do damage elsewhere. The second objection raised was that the constricting of the limb with a tube or a narrow bandage at one point was liable to do injury, first, to the muscle and secondly, to the nerve. Dr. Elliott had used Esmarch's method as modified by Dr. Senn with great satisfaction.

Dr. O. H. Buford, of Cartersville, reported a rare case in obstetric practice, showing hour glass contractions on fœtus.

Dr. W. P. Williams, of Blackshear, read a paper entitled "Persistent Remittent, or So-Called Typho-Malarial Fever." The author gave an analysis of a few cases of persistent remittent fever, touching its diagnosis and differential diagnosis from typhoid fever especially. He presented the clinical side of the matter, as his investigations extended no further. He presented three forms for consideration: *First*, the abortive form, or such as are immediately amenable to treatment. *Second*, the continued form with low temperature. *Third*, the continued form with high temperature. Under the first form, he was called to see the case of a negro that had been sick about a day. He found a temperature of 106°, no other striking symptom. He gave him fifteen grains of quinine, and left two other doses to be repeated at six-hour intervals. His diagnosis and prognosis were persistent remittent fever, with the prospect of a long siege. He returned the next morning and found that the patient was up and gone.

The value of a diagnosis between these maladies was apparent when we came to the subject of treatment. The treatment which the speaker had followed, which had proved reasonably successful in persistent remittent fever, was the anti-malarial preparation, principally cinchona and its derivatives, and arsenic, with cholagogue cathartics (most frequently calomel) at stated intervals, applying other medicines as they were indicated. This treatment, while very beneficial, in a large number of malarial cases, he would consider utterly useless and even dangerous in applying it to typhoid fever cases. The author closed his paper by saying that for every degree in rise of temperature there was a corresponding increase of heart action from eight to ten beats. In several of his cases of this fever this ratio was completely destroyed, and in some the reverse took place.

DR. M. L. CURRIE, of Mount Vernon, read a paper entitled "Periproctitis with an Abscess and Report of a Case." He said periproctitis was one of those infrequent inflammatory diseases which the general practitioner might at any time be called to treat, and which he might fail to recognize until much damage to his patient ensued. It is usually suppurative in character, but a cure may be effected by absorption, even after a distinct tumor is formed. Of the cause of the disease but little can be said. It may result from traumatism, foreign bodies, extension of adjacent inflammatory processes, or any structural disease involving the mucous membrane of the rectum. The manner of its extension and the course of the morbid processes excited, are identical with those seen in perityphlitis following typhlitis. The prognosis depends much upon the time when a diagnosis is made, the treatment of both the inflammation and the abscess, as well as the physical condition of the patient. When the vitality is low, and the abscess is high up in the pelvic cavity, or when the patient is tuberculous, it is unfavorable; when otherwise, we may hope for recovery. The author then gave the diagnostic symptoms and the treatment in connection with the case he reported.

DR. R. R. KIME, of Atlanta, followed with a paper on "Ectopic Pregnancy, its Pathology, Symptoms and Treatment." After dwelling upon the pathology and symptoms, the author summarized the treatment as follows:

1. In primary or secondary intra-peritoneal rupture of ectopic sac operate by cœliotomy at once.

2. Before primary rupture with diagnosis of tubal pregnancy extirpate cyst, tube and ovary. Fœticide for this condition by electricity and morphia injections are but temporary expedients suitable for cases of doubtful diagnosis, or where a competent operator cannot be obtained. Their use only allures patients into a false hope of security when delay is dangerous. If diagnosis could be made in first three or four weeks of pregnancy, then electricity might accomplish more potent results.

3. With extra-peritoneal primary rupture into broad ligament in early weeks of gestation producing pelvic hematocele, wait for absorption of effused blood; if it fail, then perform cœliotomy or vaginal drainage.

4. If child survives primary rupture of tube into broad ligament, do not commit murder, keep a clean conscience toward God and man, let fœtus live, keeping patient under strict observance ready to operate at any time when life of patient demands it, or fœtus at least reaches a viable period. By waiting for development of fœtus and cyst as Tait claims, the peritoneum is pushed up on the side in which they are located, so that in later months of pregnancy a lateral incision will enter cyst, and fœtus may be extracted without entering abdominal cavity.

5. Surgical interference should be the rule in all cases of ectopic pregnancy reaching full term, even after spurious labor and death of fœtus.

DR. LUTHER B. GRANDY, of Atlanta, read a paper entitled "A Board of Medical Examiners; the State's Medical Duty.' (Published in this issue.)

DR. ARTHUR C. BLAIN, of Macon, contributed a paper entitled "The Practice of Medicine in Georgia." He called attention to the want of laws governing the practice of medicine in the State. He also emphasized the importance of creating a State Board of Medical Examiners. Georgia was made a dumping-ground for all who failed to pass the requisite examination to obtain a license to practice in Virginia, Florida or Alabama. State boards not only afforded protection to the people from charlatans and unqualified practitioners, but exerted a salutary influence toward elevating the educational standard of the medical profession. The speaker recommended that a committee be sent to Atlanta during the next session of the legislature to assist in formulating and passing some good law that would meet the requirements of the case.

DR. J. C. AVARY, of Atlanta, followed with a paper on "State and Municipal Hygiene."

The three papers were ably discussed by Drs. C. D. Hurt, R. O. Engram, Willis F. Westmoreland, W. H. Elliott, F. W. McRae, M. B. Hutchins, and a committee of one from each congressional district appointed for the purpose previously outlined.

### SECOND DAY—AFTERNOON SESSION.

DR. C. D. HURT, of Atlanta, read a paper entitled "Etiology of Puerperal Eclampsia, its Treatment, with Report of Some Typical cases." After a careful study of various authors, and summing up his observations and experience at the bedside, the author felt warranted in offering the following conclusions: (1) That eclampsia gravidarum in every case is the result of some influence which is directly or indirectly exerted upon the nervous system, causing derangement of its functions. (2) That such influences may exist singly or be co-operative, and that such influence may be direct violence or mechanical pressure. (3) That one or more of these influences may reach the nervous system by absorption or through the circulation. (4) That by chemical changes wrought under certain conditions in the system, toxic elements may be created and proved deleterious. Again, certain elements are harmless as they exist in normal proportions in the constitutions, but exert a toxic influence in abnormal quantities. Urea as a toxic element, abnormally increased by retention in the system, is assigned by many as the most frequent cause. Yet there are some who do not hold to this opinion. All admit that eclampsia and uræmia coexist in a very large per cent. of cases, while they disagree as to their relation of cause and effect. Furthermore, all admit that uræmia cannot be found to exist in every case of eclampsia, therefore other causes than uræmia can and do produce eclampsia. That in every case irritation may and does exert an influence which materially aids in bringing on a seizure, and that in many cases this irritation, intensified by protracted labor, hard pains, unyielding os and exhaustion of the nervous system, develops eclampsia. That a plethoric condition of the patient, full habit, distended blood vessels increased susceptibility to an apoplectic condition and excessive irritability, and that with this plethoria a crowding of the blood into the lungs and upon the brain, materially interfere with the functions of the nervous system. That the emotional system is not silent in some women, and that it helps to precipitate a fit.

DR. H. MCHATTON, of Macon, read a paper entitled "Four Women who Refused Oöphorectomy, and their Subsequent Histories."

The author gave a brief *résumé* of the histories of the only four women that he had ever known who refused the operation. The time that had elapsed since it was advised varied from eighteen months to twelve years. The speaker made the point that in each case the operation was advised and urged by gynecologists of standing both North and South, consequently they must have been convinced that they were cases demanding operative interference. In a practice of twelve years he had had occasion to recommend the removal of the appendages once, excepting cases of ovarian tumors, and that one proved to be a case of pyosalpinx. Several of his patients had drifted into other hands and had oöphorectomy performed, and as far as he could learn had been disappointed in the results each time. The best men in this special line of work were doing the operation less and less each year. Their place was being amply filled by lesser lights, with smaller numbers of individual cases, but with a yearly aggregate that is terrible to contemplate. By what combination of circumstances could one man, to fame unknown, in a small interior city, and in a short space of time, find 144 cases demanding abdominal section?

DR. C. H. PEETE, of Macon, contributed a paper on "Partial Tenotomy, a Radical Cure for Heterophoralgia." The author finds that the majority of cases suffering from heterophoralgia are those of young adult life, or that they have been suffering since that time. He accounts for it by the fact that we know in order to cause it the patient must concentrate his vision on an object, and the length of time of this concentration governs the intensity of suffering. In children the vision is never fixed on any point for a length of time, consequently the symptoms are not brought on. In young adults who have acquired the habit of hard study and constant close work, it occurs frequently, and we will find in most cases that it commenced with the time of their beginning to work, or in a short time after. The muscle tests should be applied to every case that comes to the oculist for refraction work, and when he finds these deviations existing, he should resort to the radical relief, partial tenotomy.

DR. F. W. MCRAE, of Atlanta, read a paper entitled "Stone in the Bladder," with report of cases. He dealt with the etiology, symptomatology and treatment of stone in the bladder, purposely avoiding extensive details and references to authorities. The chief predisposing causes were defective digestion and assimilation, due either to improper diet, to a preponderance of solid over liquid ingesta, or to too high living coupled with insufficient exercise and imperfect oxidation. There is an excess of the solids of the urine. These are only predisposing causes, and others, such as mucus, pus, etc., due to inflammatory conditions of the urinary mucous membrane, must be present before stone in the bladder will result. A history of nephritic colic not followed by the expulsion of the gravel through the urethra, would naturally lead us to suspect the formation of stone in the bladder when followed by irritation of that viscus. Only when the stone can be felt by the searcher and the characteristic click elicited are we sure of the presence of calculus in the bladder. In one of the cases which the author reported the symptoms were characteristic of stone in the bladder, although no stone was found. The operation was a brilliant success in relieving the patient of a most violent cystitis of long standing. The author then dealt with the methods of treating stone in the bladder, and said that no method had so wide a range of applicability and as low a death rate as litholapaxy (Bigelow's operation). He had had, however, no personal experience with it, as the cases thus far presenting themselves to him were such as might be best treated by other procedures.

This operation offered much the best results except in very young children, very large or very hard stones, or where there was co-existing tight organic strictures or enlarged prostate of such character as to prevent the introduction of the lithotrite, or where there was a violent cystitis associated with stone. For large stones associated with prostate or vesical tumors the high operation was undoubtedly far superior to the perineal operations. Small stones associated with stricture of the deep urethra or violent cystitis were best treated by the median operation. Nor was the lateral operation to be entirely set aside for the now more popular operations of litholapaxy and supra-public lithotomy. Each of these operations had its proper field of usefulness, and it was only by a careful selection of the methods of treatment that the best results were to be obtained.

DR. W. R. GOOGE, of Abbeville, reported a case of fracture of the skull, with protrusion of the brain substance and removal of same.

THIRD DAY—MORNING SESSION.

DR. J. B. S. HOLMES, of Rome, read a lengthy paper on "The Technique and After-treatment of Ovariotomy." The author offered the paper as an *exposé* of the method adopted and successfully used by him in his abdominal work. He submitted the paper with the earnest hope that it might elicit a full and free discussion from all those interested in this line of work. By such discussions our methods may be compared, and those adopted that promise best results to our patients. No one could truthfully say that in every particular his method was the best. While we might in the main agree, yet upon slight and seemingly indifferent details might hinge the successful issue of our cases. He stood ready and willing to take up any suggestions from his brother surgeons if he could get better results by so doing. The author then took up each step *seriatim* of the technique and after-treatment.

DR. FRANK M. RIDLEY, of LaGrange, then delivered the orator's address. He selected for his subject "Woman's Relations to the Practice of Medicine."

DR. M. B. HUTCHINS, of Atlanta, contributed a paper entitled "Mechanical Treatment of Some Skin Anomalies." The author dwelt upon some of the various mechanical means of removing anomalies of the skin. The destruction of superfluous hairs, of moles, birth-marks, and the various simple growths of the skin were described. In 1879 Dr. Michel, of St. Louis, successfully employed electrolysis for the destruction of the hairs in trichiasis. Dr. Hardaway, a dermatologist of the same city, followed the idea in the treatment of hypertrichosis. Various dermatologists had since reported upon its successful use. Hair upon the lip and face of women afforded the usual field of operation.

Three to eight cells of a galvanic battery were used. For the past two years Dr. Hutchins had used some "Law" cells put together by himself, containing as the fluid an aqueous solution of sal ammoniac. For the destruction of hairs five of these were sufficient, though they had not been filled in two years. Five to seven of these cells sufficed for ordinary moles. To the negative pole a pencil shaped needle holder, containing a number eight or twelve ordinary sewing needle, is attached, while the positive pole is provided with the usual sponge electrode. The needle is attached to the negative pole for the reason that electrolysis through the positive will leave a black dot at the point of insertion of the needle. The author prefers the needle holder having a spring for opening or closing the circuit, as this leaves the entire control of the circuit to him, the patient steadily holding the well-wetted sponge electrode in one hand instead of having to open and close the circuit by letting go or catching hold of the sponge. The needle is carefully inserted into the hair follicle, parallel to the root, until slight resistance, the bottom of the follicle and the seat of the hair papilla, is felt. The circuit is then closed and the current allowed to pass until a frothy substance the size of a small pin head fills the follicle, and a small area of blanching appears around the needle. If the needle has followed the course of the hair root and remained in the follicle, the pain is less than when the root sheaths are pierced, and the frothy substance is more abundant. If the hair is easily pulled out it is reasonably certain that there will be no regrowth, but if it pulls out with difficulty or breaks off, the current must be immediately reapplied. A pair of forceps made for that purpose are used to extract the hairs.

Dr. Hutchins then reported several interesting cases which he had treated successfully by electrolysis.

The following officers were elected:

President—Dr. W. H. Elliott, Savannah.

First Vice-President—Dr. G. T. Miller, Americus.

Second Vice-President—Dr. H. McHatton, Macon.

Secretary—Dr. Dan H. Howell, Atlanta.

Treasurer—Dr. E. C. Goodrich, Augusta.

The next annual meeting of the association will be held in Atlanta.

